# Apoptosis and Pathogenesis of Avian Influenza A (H5N1) Virus in Humans

**DOI:** 10.3201/eid1305.060572

**Published:** 2007-05

**Authors:** Mongkol Uiprasertkul, Rungrueng Kitphati, Pilaipan Puthavathana, Romchat Kriwong, Alita Kongchanagul, Kumnuan Ungchusak, Suwimon Angkasekwinai, Kulkanya Chokephaibulkit, Kanittar Srisook, Nirun Vanprapar, Prasert Auewarakul

**Affiliations:** * Mahidol University, Bangkok, Thailand; † Ministry of Public Health, Bangkok, Thailand; ‡ Phaholpolphayuhasena Hospital, Kanjanaburi, Thailand

**Keywords:** Avian influenza A virus H5N1, autopsy, apoptosis, pathogenesis, lymphopenia, lung, spleen, intestine, tumor necrosis factor-α, research

## Abstract

The pathogenesis of avian influenza A (H5N1) virus in humans has not been clearly elucidated. Apoptosis may also play an important role. We studied autopsy specimens from 2 patients who died of infection with this virus. Apoptosis was observed in alveolar epithelial cells, which is the major target cell type for the viral replication. Numerous apoptotic leukocytes were observed in the lung of a patient who died on day 6 of illness. Our data suggest that apoptosis may play a major role in the pathogenesis of influenza (H5N1) virus in humans by destroying alveolar epithelial cells. This pathogenesis causes pneumonia and destroys leukocytes, leading to leukopenia, which is a prominent clinical feature of influenza (H5N1) virus in humans. Whether observed apoptotic cells were a direct result of the viral replication or a consequence of an overactivation of the immune system requires further studies.

## Abstract 2

Apoptosis may play a crucial role in the pathogenesis of pneumonia and lymphopenia caused by this virus in humans.

The pathogenesis of avian influenza A (H5N1) virus in humans is not well understood. Although several studies have shown some aspects of this pathogenesis in animal models, direct evidence of pathogenic mechanisms in humans has been limited to only a few autopsy studies (*1*–*3*). We previously demonstrated in an autopsy case that alveolar epithelial cells are the major target cell type of this virus (*3*). The case in that study, as well as other previous autopsy reports, died late in the disease. Some of the findings may not reflect the actual pathogenesis at the acute period but may be consequences of secondary events. We performed an autopsy of a patient who died on day 6 of onset of illness. The findings in this case are more likely to reflect viral pathogenesis in the acute phase of the disease.

Apoptosis has been implicated in the pathogenesis of influenza. Infection of epithelial cells and lymphocytes has been shown to induce apoptosis in vitro (*4*–*8*). Several modes of apoptosis induction and responsible viral genes have been proposed (*8*–*13*). Infection with virulent influenza (H5N1) virus was also shown to induce lymphopenia and lymphocyte apoptosis in vivo (*14*). However, whether and to what extent apoptosis contributes to the highly virulence property of influenza (H5N1) viruses are not clear. In this report, we studied apoptotic activity in 2 patients who died of avian influenza.

## Materials and Methods

### Patients

The study was approved by the Siriraj Ethics Committee. The first patient (patient A) was a 48-year-old man who had progressive viral pneumonia. He had fever, cough, running nose, myalgia, and chest pain at the onset of illness. Dyspnea developed on day 2 of illness, and a chest radiograph showed interstitial infiltrations at right upper and left middle lung fields and a masslike infiltration at the right middle lung field. The diagnosis of avian influenza was suspected on day 4 of illness after a history of direct contact with dying chickens was revealed. Respiratory secretions were then sent to national laboratories and confirmed positive for influenza (H5N1) virus. The patient died on day 6 of illness.

An autopsy was conducted by using standard techniques and precautions to minimize risk for transmission of infection. Tissues obtained were prepared for routine histologic analysis and samples were stored at −70°C for further study.

The other autopsy case (patient B) has been previously reported (*3*). This patient was a 6-year-old boy who had progressive viral pneumonia that led to acute respiratory distress syndrome and death 17 days after onset of illness.

### RNA, Antigen, and Apoptosis Analyses

Lung, trachea, liver, spleen, colon, and bone marrow tissues were tested for viral RNA. For reverse transcription–PCR (RT-PCR), fresh unfixed specimens were minced into small pieces in lysis buffer of an RNA extraction kit (RN easy; QIAGEN, Valencia, CA, USA). Total RNA was then extracted according to the manufacturer’s protocol. RT-PCR for hemagglutinin 5 (H5) was then performed on the extracted RNA by using the One-Step RT-PCR Kit (QIAGEN) with an H5-specific primer pair. Strand-specific RT-PCR was performed by using a method similar to the RT-PCR for viral RNA detection except that only 1 primer was added at the reverse transcription step. Tumor necrosis factor-α (TNF-α) mRNA was detected in RNA extracted from lung, trachea, liver, spleen, colon, and bone marrow tissues by an RT-PCR as previously described (*3*).

Tissue sections of lung, trachea, liver, spleen, and colon were stained for influenza A virus antigen. The sections were deparaffinized and rehydrated. Antigenic sites were identified by digestion with 0.5% trypsin for 15 min at 37°C. Endogenous peroxidase activity was blocked by incubating sections in 3% H_2_O_2_ for 15 min at 37°C. Sections were incubated with 2.5% bovine serum albumin (Dako, Roskilde, Denmark) for 15 min at room temperature and subsequently incubated with a monoclonal antibody to influenza A virus nucleoprotein at a dilution of 1:40 (B.V. European Veterinary Laboratory, Woerden, the Netherlands) overnight at 4°C. Slides were rinsed 3 times in 1× phosphate-buffered saline (PBS) plus 0.05% Tween-20 and incubated with horseradish peroxidase–conjugated goat antimouse immunoglobulins at dilutions of 1:400 (Dako) for 30 min at room temperature. The slides were washed as above and developed with diaminobenzidine (Dako).

Lung, liver, spleen, colon, and bone marrow sections were analyzed for apoptosis by using the terminal deoxynucleotidyl transferase-mediated dUTP-biotin nick end-labeling (TUNEL) assay. After digestion with 0.5% trypsin as described above, sections were treated with TUNEL reaction mixture by using the In Situ Cell Death Detection Kit (Boehringer, Mannheim, Germany) for 1 h at 37°C in the dark. Slides were then rinsed 3 times with 1× PBS and incubated with alkaline phosphatase–conjugated fluorescein isothiocyanate–labeled antibody for 30 min at 37°C. Sections were then washed and developed with nitro-blue tetrazolium chloride/5-bromo-4-chloro-3-indolyl phosphate p-toluidine.

## Results

### Pathologic Findings

**Figure 1:**
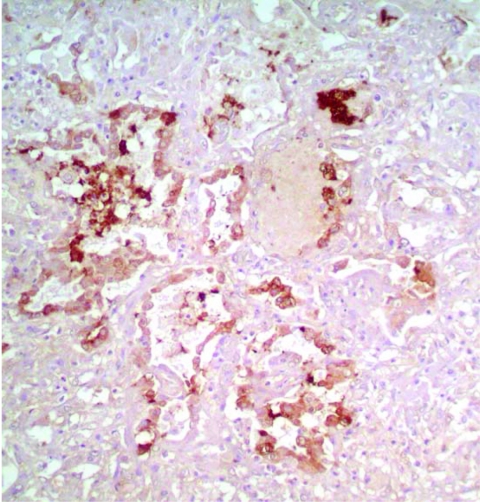
Immunohistochemical staining of viral antigen in alveolar epithelial cells of patient A infected with avian influenza A (H5N1) virus (magnification ×100).

Pathologic findings in patient A were not identical to the previously reported findings in patient B (*3*). Common findings in both patients were diffuse alveolar damage and positive staining for influenza A virus antigen in alveolar epithelial cells (Figure 1). In patient A, lungs showed an earlier stage of exudative phase of diffuse alveolar damage than the damage found in patient B. Patient A also showed some atypical pneumocytes with large bizarre and clumping nuclei. Bronchiolitis and pleuritis were also observed. No superimposed bacterial or fungal infection was identified. Hemophagocytic activity was found in lungs, liver, and bone marrow. The liver showed some cholestasis but was otherwise unremarkable.

### Sites of Viral Replication

**Figure 2:**
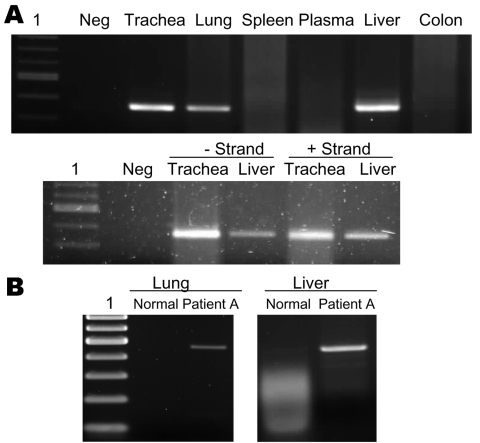
A) Detection of viral RNA in lung, trachea, and liver by reverse transcription–PCR (RT-PCR) (upper panel) and detection of positive- and negative-stranded viral RNA in trachea and liver by strand-specific RT-PCR (lower panel). Lane 1, 100-bp ladder; Neg, negative. B) RT-PCR showing overexpression of tumor necrosis factor-α in lung and liver tissues of patient in A compared with normal tissues.

Viral RNA was detectable in the lung, trachea, and liver of patient A (Figure 2A). To test whether virus replicated outside the lung, we tested the trachea and liver by using a strand-specific RT-PCR and found that both tissues contained positive-stranded viral RNA, which suggested active viral replication in these organs. This was in contrast to our previous report (patient B), in which positive-stranded viral RNA was detectable in lung and intestine (*3*). Whether this was a result of a difference in tissue tropism of the viruses is unclear. It is also possible that this reflected different phases of the disease course between the 2 patients. The finding also indicated that viral replication was maintained in the lung throughout the course of the disease. This was in agreement with the finding in both patients that viral antigen was detectable by immunohistochemical analysis only in alveolar epithelial cells of the lungs, which indicated that this cell type is the major target cell in humans.

### Apoptosis

**Figure 3:**
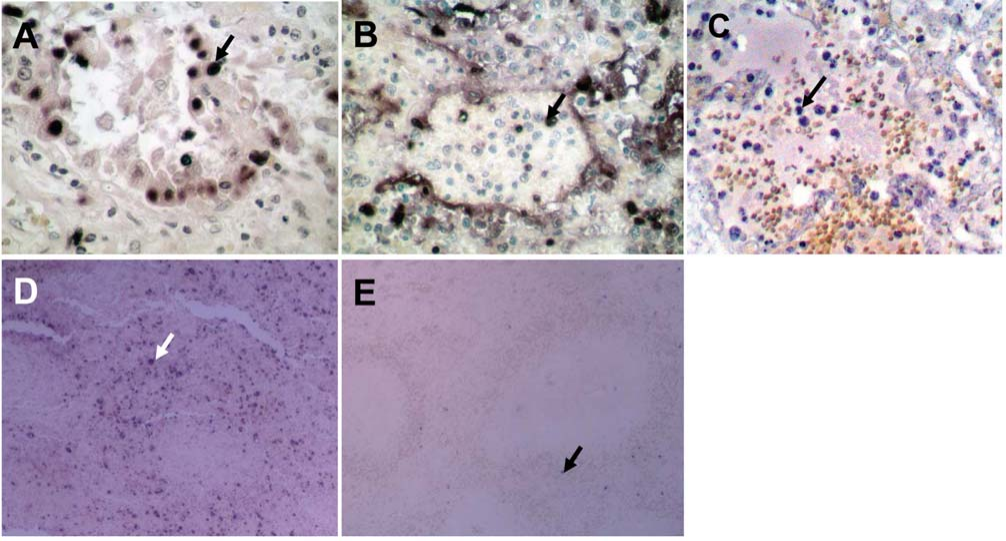
Terminal deoxynucleotidyl transferase–mediated dUTP-biotin nick end-labeling staining showing numerous apoptotic alveolar epithelial cells in lung of patient B (A) and leukocytes in lung of patient A (B). C) Lung tissue from a patient with pneumonia caused by human influenza A (H5N1) virus showing apoptosis only in leukocytes. D) Spleen of patient B showing numerous apoptotic cells. E) Normal spleen tissue showing only a minimal level of apoptosis. Apoptotic cells are stained dark blue and an apoptotic cell in each panel is indicated by an arrow. Magnification ×400 in A, B, and C; ×100 in D and E.

Apoptosis was frequently observed in hyperproliferating alveolar epithelial cells (Figure 3A) in lung tissues from patient B. In patient A, apoptosis was prominent in leukocytes that infiltrated the lung, but apoptotic alveolar epithelial cells were less frequent partly because epithelial cells were mostly absent, which left alveolar surface denuded (Figure 3B). We have previously shown that TNF-α mRNA was upregulated in the lung of patient B (*3*), which may be 1 of the mechanisms leading to apoptosis. Similarly, we tested the lung of patient A and found upregulation of TNF-α mRNA. We also tested trachea, liver, spleen, colon, and bone marrow tissues of this patient; TNF-α mRNA was detectable only in the liver (Figure 2B).

To compare these findings with those of viral pneumonia caused by human influenza virus, we searched archival pathologic specimens for lung tissues with histopathologic findings compatible with viral pneumonia. The specimens were then tested for influenza A virus by RT-PCR. A virus-positive specimen was stained for apoptosis. This lung specimen was from a 47-year-old man who sought medical attention because of fever and hemoptysis. A lung biopsy was performed to investigate the cause of hemoptysis. Pathologic examination showed bronchiectasis and interstitial pneumonia. The patient responded to supportive care and recovered. In comparison with lungs infected with influenza (H5N1) virus, no apoptotic alveolar epithelial cells were detected in this lung tissue that contained human influenza virus. However, apoptotic leukocytes infiltrating the lung were as prominent as in patient A (Figure 3C).

Apoptotic lymphocytes were abundant in the red pulp and occasionally observed in the white pulp (Figure 3D) of the spleens of both patients. In contrast, a normal spleen specimen showed only a minimal number of apoptotic cells (Figure 3E). We also observed apoptotic cells in intestinal epithelial cells of patients A and B. Liver samples from patient A did not show large numbers of apoptotic cells despite the presence of replicating viral RNA and TNF-α mRNA. The lack of apoptosis in the liver and other organs and cell types indicated that the observed apoptotic cells in lungs, spleens, and intestines were specific to the pathologic process and not due to postmortem changes.

Because leukopenia and thrombocytopenia are prominent clinical features of infection with influenza (H5N1) virus (*15*), we investigated whether bone marrow failure plays a role in addition to increased destruction by apoptosis of leukocytes in the lung. We stained bone marrow samples from both patients for a proliferation marker, Ki-67, and an apoptotic marker. These samples showed normal levels of Ki-67+–proliferating cells when compared with normal bone marrow samples. The number of apoptotic cells in bone marrow did not increase. This finding suggested that bone marrow may maintain normal function during infection with influenza (H5N1) virus and is likely not responsible for the leukopenia and thrombocytopenia.

## Discussion

Loss of alveolar epithelial is probably 1 of the pathogenic mechanisms of pneumonia caused by influenza (H5N1) virus, and apoptosis is at least partly responsible. The hyperproliferation of pneumocytes observed in patient B was likely the regenerative process in the late phase of the disease. Whether the apoptosis of alveolar epithelial cells was a direct result of infection in those cells or an indirect consequence caused by cytokine dysregulation is not yet clear. Expression of influenza viral genes has been shown to induce apoptosis in infected cells (*8*–*13*). These viral genes likely play a role in induction of apoptosis. However, the role of cytokines in apoptosis in pneumocytes cannot be excluded.

Lymphopenia has been shown to be a predictive marker for acute respiratory distress syndrome and death (*15*). Infection of primary human lymphocytes in vitro has been shown to induce apoptosis (*7*). Increased apoptosis leading to severe lymphopenia was likely evidence of more active viral replication and higher viral load. Because we did not find evidence of viral replication in the spleen of patient A, which contained many apoptotic cells, lymphocyte apoptosis may not be a direct consequence of infection in these cells. Apoptosis of lymphocytes may have been caused indirectly by cytokine dysregulation and overactivation of the immune response.

Because we also found numerous apoptotic leukocytes in lungs of a person infected with influenza virus and leukopenia is not a prominent clinical feature of human influenza, apoptosis of infiltrating leukocytes in inflamed tissue alone may not be sufficient to cause leukopenia. Conversely, systemic cytokine dysregulation during infection with influenza (H5N1) virus may cause massive apoptosis in lymphoid organs, which leads to lymphopenia. Another possible explanation is that apoptosis was induced locally while lymphocytes were circulating through the infected lung, Determining if apoptotic lymphocytes can be directly detected in patients’ blood and can be a predictive marker for disease outcome requires further studies.

Apoptosis could not be detected in the liver despite the presence of viral RNA and TNF-α mRNA. The liver did not show severe inflammation as observed in the lung, which suggests that without apoptosis viral infection would not cause severe tissue damage. This finding underscores the role of apoptosis in viral pathogenesis. Conversely, absence of apoptotic cells in the liver may indicate that viral replication in this organ was not sufficient to induce apoptosis and pathologic changes. The absence of apoptosis in the liver, despite the presence of TNF-α mRNA, suggests that expression of TNF-α alone may not be the major mechanism responsible for induction of apoptosis in pathogenesis of influenza virus.

We did not detect viral antigen in other organs, despite the presence of viral RNA. This finding suggested that although other cell types were permissive for replication of viral RNA, this replication in these cells was inefficient. This is in contrast to the widespread presence of viral antigen in animal tissues and probably reflects the interspecies barrier and incomplete adaptation of influenza (H5N1) virus to the human host. Although a recent reported showed that upper airway epithelium of humans lacks the α-2,3–linked sialic acid receptor for avian influenza virus (*16*), other in vitro data suggest that α-2,3–linked sialic acid is expressed in ciliated columnar epithelial cells of the airway (*17*,*18*). Recent in vitro experiments showed that the α-2,3–linked sialic acid receptor specific for avian influenza virus replicates and spreads poorly in cultured differentiated human tracheobronchial epithelial cells (*19*,*20*). This finding is consistent with our data, which showed no detectable viral antigen in the trachea and probably reflects inefficient infection of influenza (H5N1) virus in this tissue.
